# Supplementary material for: The genetics of an early Neolithic pastoralist from the Zagros, Iran
Source: Sci Rep. 2016 Aug 9;6:31326. doi: 10.1038/srep31326 (PMC4977546; doi:10.1038/srep31326)
Supplement: Supplementary Information [file srep31326-s1.pdf]

Supplemental Information for:

The genetics of an early Neolithic pastoralist from the Zagros, Iran

Authors: Gallego-Llorente, M., Connell, S., Jones, E. R., Merrett, D. C., Jeon, Y., Eriksson, A., Siska, V., Gamba, C., Meiklejohn, C., Beyer, R., Jeon, S., Cho, Y. S., Hofreiter, M., Bhak, J., Manica, A., Pinhasi, R.

S1. Archaeological Information

Ganj Dareh Tepe, a small mound in the Gamas-Ab Valley, is at an altitude of ~1400 m at the entrance to a small side valley of the High Zagros Mountains in Kermanshah Province, Western Iran. The site, situated close to a protective wall of limestone[1], is one of several discovered during survey work in the area[2]. The Tepe, measuring ~40 m in diameter, has five occupational levels in the 7 to 8 m of cultural deposits, levels A to E, with level E at the base. Permanent mud brick architecture is first seen in level D. After initial sondage in 1965, Philip E.L. Smith excavated roughly 20 percent of the mound in four seasons between 1967 and 1974. Zooarchaeological evidence shows a population exploiting ovicaprids, with goat dominant. In contrast the archaeobotanical evidence provided small numbers of both wild (shattering, brittle-rachised) and domesticated (non-shattering, tough-rachised) morphological forms of barley[3]. Inferences at the time suggested limited use of domesticated barley at Ganj Dareh. However, since both morphological forms are expected in wild barley[4], small proportions of tough-rachised barley internodes are no longer considered definitive evidence for use of domesticated barley[5]. No other cereals were recovered from the site[3]. There is evidence for herding but no evidence for decreased size or changes in horn core morphology[6,7]. Current evidence places the occupation of the site at ca 8950-8700 bp or 9950-9650cal BP[7]. None of the human remains have been directly dated.

The individual analysed here is adult GD#13A, a 30-50 year-old female. The left petrous was sampled (Fig. S1A). This was also one of the individuals recognized by Meiklejohn et al. in 1992[8] as showing cranial deformation (Fig. S1B).

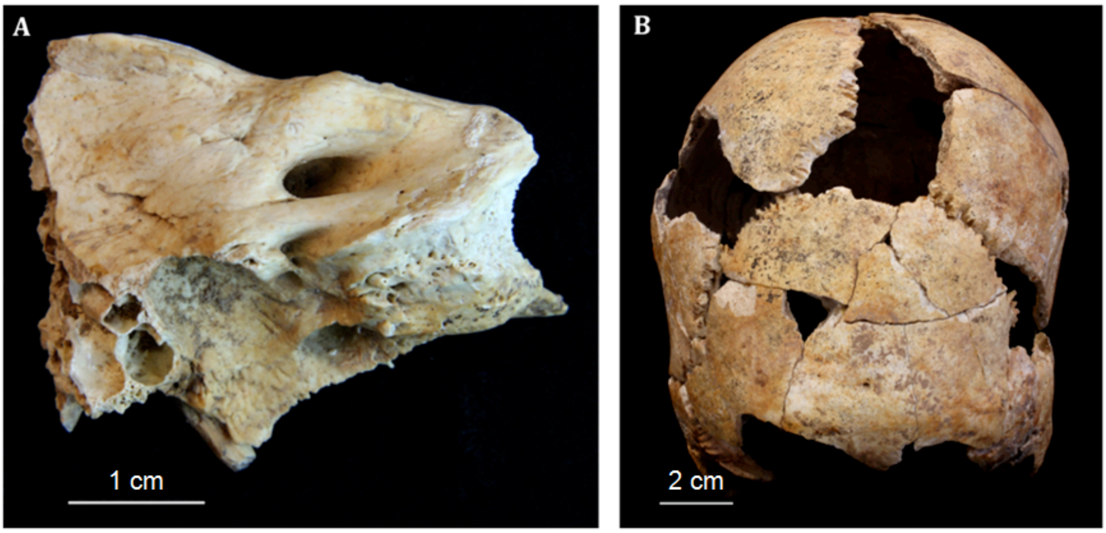

Fig. S1. GD13A skeletal remains. A) Medio-inferior view of the petrous portion of the temporal bone used for DNA extraction. **B)** Posterior view of reconstructed skull of the GD13a individual showing cranial deformation.

S2. Sequence processing and alignment

Table S1. Alignment statistics for GD13a.

Sample	Total reads	Aligned reads	(%)	High quality reads	(%)	Coverage (x)
GD13a	728,931,167	135,327,301	18.57	90,189,417	12.37	1.39

Libraries from GD13a were sequenced over a flow cell on a HiSeq 2000 using 100bp single-end sequencing. Aligned reads refer to non-duplicated sequences while high quality reads includes non-duplicated sequences with mapping quality ≥ 30 and length ≥ 30.

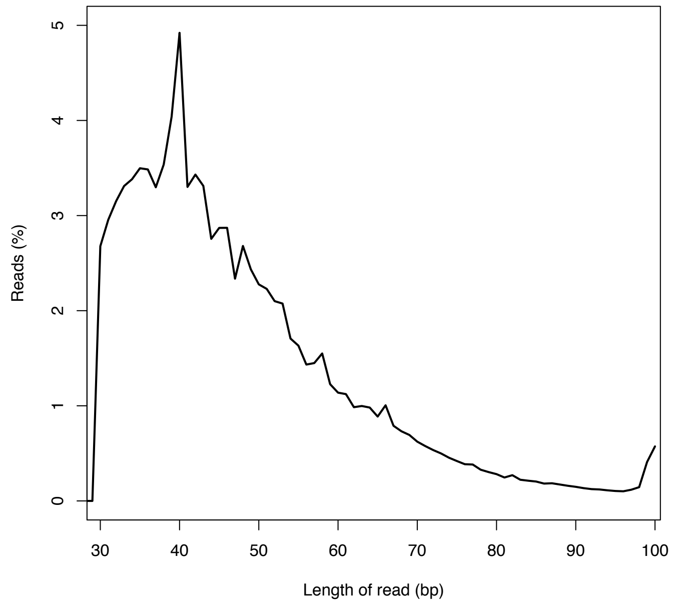

Fig. S2. Sequence length distribution for GD13a.

S3. Authenticity of results

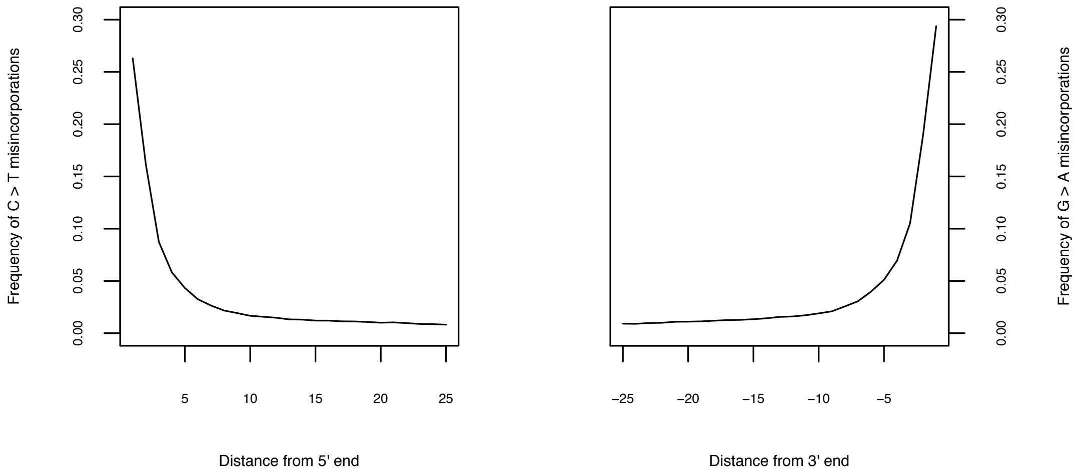

Fig. S3. Damage patterns for GD13a. Plots show mismatch frequency relative to the reference genome as a function of read position. The left hand figure shows the frequency of C to T misincorporations at the 5’ ends of reads (first 25 bases) while the right hand figure shows the frequency of G to A transitions at the 3’ ends of reads (last 25 bases).

S4. Principal component analysis shows that Southern Asian populations are the closest contemporary populations to the Iranian herder

GD13a was placed close to the Southern Asian samples, specifically between the Balochi, Makrani and Brahui populations of South Asia. (Fig. S4). Of the ancient samples, GD13a falls closest to hunter-gatherers from the Caucasus (Fig. S4).

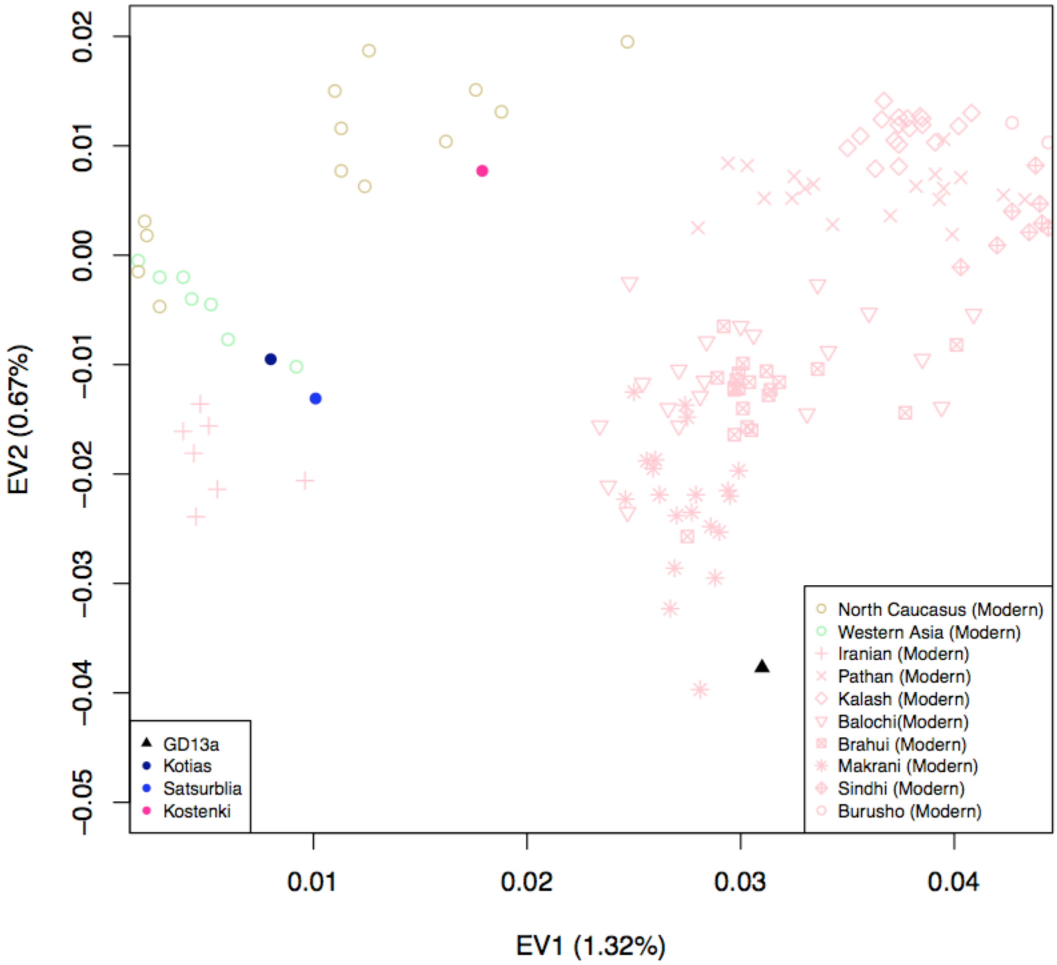

Fig. S4. PCA with ancient individuals projected onto principal components 1 and 2 which are defined by modern populations. Here we zoom (full dataset shown in Fig. 1) into the populations close to GD13a, revealing affinities to modern Balochi, Brahui and Makrani populations.

S5. ADMIXTURE shows that the Iranian herder shares a large part of its genome with Caucasus Hunter Gatherers, with a small proportion of southern Asian-like alleles

In Fig. S6, samples are hierarchically clustered by region and populations. For the sake of clarity, ancient samples are positioned on the left side of the figure and represented as bars with a width corresponding to five individuals. There are no clear outliers in any population, suggesting that they were well-defined and that the number of SNPs was sufficient to correctly define clusters, even after LD-based pruning.

The cluster membership of published modern and ancient samples is similar to previous analyses[9,10]. GD13a harbours mainly the “green” component found in Caucasus Hunter-Gatherers. This is in agreement with our PCA analysis, as well as the geographic proximity of Iran to the Caucasus. Only modern populations which have the “green” component found in GD13A are shown in Fig. 1c. In addition to this component, GD13a also harbors a small component found in modern southern Asian populations.

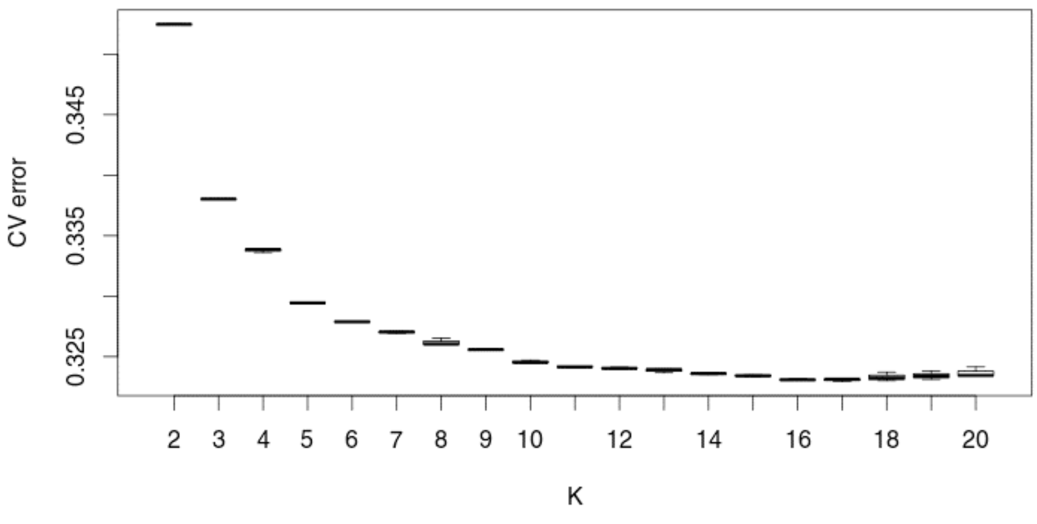

Fig. S5. ADMIXTURE analysis cross validation (CV) error as a function of the number of clusters (K). Both the lowest minimal and mean value was attained at K=17.

[Figure S6 attached as a PDF as it is too large to fit here]

Fig. S6. ADMIXTURE analysis for 2-20 clusters (K).

S6. Outgroup f_3 statistics show that GD13a shares the most genetic drift with Caucasus Hunter-gatherers

We used outgroup f_3-statistics to estimate the amount of shared drift between GD13a and contemporary populations. We computed $f_3(X$, GD13a; Dinka) and $f_3(X$, GD13a; Ju'Hoansi South), where X represents a modern population and Dinka, an African population equally related to Eurasians, acts as an outgroup (Fig. S7). We also repeated this analysis where X represents ancient individuals/populations. Among the ancient populations, Caucasus hunter-gatherers (Kotias and Satsurblia) have the closest affinity to GD13a (Table S2 and S3), followed by other ancient individuals from Bronze Age Steppe populations and modern populations from the Caucasus.

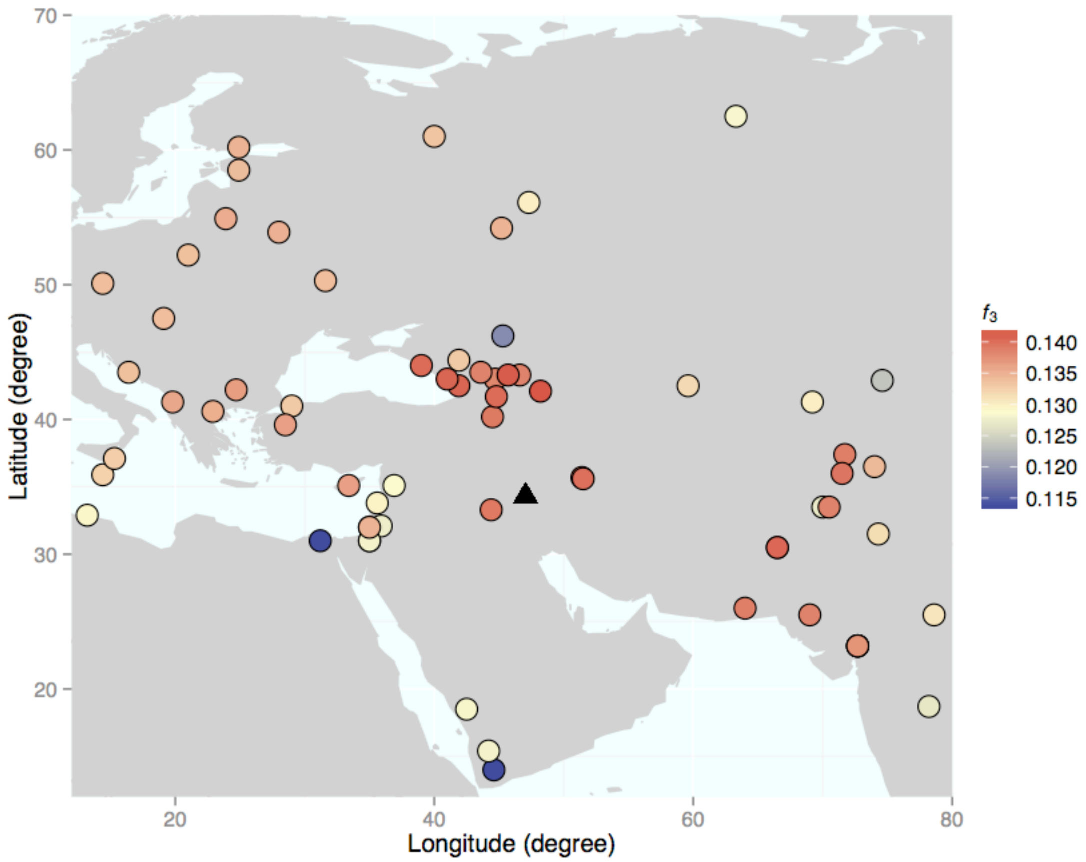

Fig. S7. ***f_3*(*X*, GD13a; Dinka) shows that the closest modern populations to GD13a are Caucasus populations and, to some extent, South Asian populations such as Balochi and Makrani. Map of populations was generated with the library "ggplot2" with R software (v3.1.2, https://cran.r-project.org/)[11]**

Table S2. f_3(*X*, GD13a; Dinka) where X represents a modern or ancient individual/population. Ancient individuals/populations are shown in bold. Populations/individuals with the largest f_3 values are shown.

X	f_3	Standard Error
**Kotias**	**0.152**	**0.003**
**Satsurblia**	**0.150**	**0.004**
**Russia (Early Bronze Age)**	**0.142**	**0.007**
**Yamnaya Samara**	**0.142**	**0.003**
Lezgin	0.142	0.002
**Unetice (Early Bronze Age)**	**0.141**	**0.003**
**Afanasievo**	**0.141**	**0.003**
Chechen	0.141	0.002
Abkhasian	0.141	0.002
Georgian	0.141	0.002
Balochi	0.140	0.002
**Corded Ware Germany**	**0.140**	**0.003**
Georgian Jew	0.140	0.002
Adygei	0.140	0.002
**Bell Beaker Germany**	**0.140**	**0.006**
**Yamnaya Kalmykia**	**0.140**	**0.003**
Iranian	0.140	0.002
Brahui	0.140	0.002
Iranian Jew	0.140	0.002
Kalash	0.140	0.002
Armenian	0.139	0.002
Iraqi_Jew	0.139	0.002
**Srubnaya**	**0.139**	**0.003**
**Irish (Bronze Age)**	**0.139**	**0.003**
Tajik_Pomiri	0.139	0.002
**Nordic (Middle Neolithic)**	**0.139**	**0.006**
Makrani	0.139	0.002
Pathan	0.139	0.002
Kumyk	0.139	0.002
Balkar	0.138	0.002
Sindhi	0.138	0.002

Table S3. f_3(*X*, GD13a; Ju'Hoansi) where X represents a modern or ancient individual/population. Ancient individuals/populations are shown in bold. Populations/individuals with the largest f_3 values are shown.

X	**f_3**	**Standard Error**
**Kotias**	**0.247**	**0.004**
**Satsurblia**	**0.243**	**0.005**
**Spain (Middle Bronze Age)**	**0.237**	**0.006**
**Corded Ware Germany**	**0.236**	**0.008**
Chechen	0.236	0.003
Abkhasian	0.236	0.003
Lezgin	0.236	0.003
Georgian Jew	0.236	0.003
Balochi	0.236	0.003
**Yamnaya Samara**	**0.236**	**0.003**
**Yamnaya Kalmykia**	**0.236**	**0.004**
**Unetice (Early Bronze Age)**	**0.235**	**0.003**
Brahui	0.235	0.003
Adygei	0.235	0.003
Georgian	0.235	0.003
Iranian Jew	0.235	0.003
Iranian	0.235	0.003
**Bell Beaker Czech**	**0.235**	**0.005**
Iraqi Jew	0.235	0.003
Kalash	0.235	0.003
Tajik Pomiri	0.234	0.003
Makrani	0.234	0.003
Pathan	0.234	0.003
**Afanasievo**	**0.234**	**0.003**
Armenian	0.234	0.003
**Andronovo**	**0.234**	**0.003**
**Battle Axe Sweden**	**0.234**	**0.005**
Sindhi	0.234	0.003
**Bell Beaker Germany**	**0.234**	**0.006**
**Samara (Eneolithic)**	**0.234**	**0.004**
Scottish	0.234	0.003

S7. *D*-statistics show that a large number of Western Eurasian samples (both modern and ancient) share higher genetic affinity to Caucasus Hunter-Gatherers than toGD13a.

We used *D*-statistics of the form *D*(Dinka, *X*; GD13a, Kotias) to investigate whether GD13a and Caucasus Hunter-Gatherers form a clade to the exclusion of other ancient and modern samples.

For all Western Eurasian populations and ancient individuals for which a difference could be detected, Kotias shows closer affinity than GD13a (Table S4).

Table S4. *D*-statistics of the form *D*(Dinka, *X*; GD13a, Kotias) where *X* represent a modern or ancient individual/population.

X	*D*-statistic	*Z*-score
**Satsurblia**	**0.090**	**6.70**
**Esperstedt_MN**	**0.058**	**4.56**
**Alberstedt_LN**	**0.057**	**4.81**
**Corded_Ware_Estonia**	**0.055**	**3.67**
**Srubnaya_Outlier**	**0.053**	**4.10**
**Halberstadt_LBA**	**0.053**	**4.56**
**Corded_Ware_Germany**	**0.050**	**5.92**
**BenzigerodeHeimburg_LN**	**0.050**	**3.83**
**Bell_Beaker_Germany**	**0.049**	**2.55**
**Afanasievo.**	**0.049**	**5.28**
**Iberia_Mesolithic**	**0.048**	**4.02**
**Samara_HG**	**0.047**	**3.04**
**Sintashta_MBA**	**0.047**	**4.56**
Georgian	0.046	6.99
**Unetice_EBA**	**0.046**	**4.51**
Orcadian	0.046	6.82
**Iberia_Chalcolithic**	**0.045**	**5.01**
**Poltavka**	**0.045**	**4.71**
**Karelia_HG**	**0.044**	**3.70**
**Bell_Beaker_Germany**	**0.043**	**4.99**
**Loschbour**	**0.043**	**4.09**
**Iberia_EN**	**0.043**	**4.48**
**MA1**	**0.043**	**3.20**
Estonian	0.042	6.11
Abkhasian	0.042	6.23
**Yamnaya_Kalmykia**	**0.042**	**4.69**
Ukrainian	0.042	6.11
Croatian	0.041	6.10
**Yamnaya_Samara**	**0.041**	**5.07**
Czech	0.041	5.91
Norwegian	0.040	5.85
English	0.040	5.75
**Remedello_BA**	**0.040**	**3.12**
French_South	0.040	5.51
Lithuanian	0.040	5.79
**Baalberge_MN**	**0.039**	**2.70**
Kumyk	0.039	5.75
Hungarian	0.039	5.83
**Srubnaya**	**0.039**	**4.92**
Icelandic	0.039	5.60
North_Ossetian	0.039	5.73
French	0.038	5.80
Adygei	0.038	5.74
**BattleAxe_Sweden**	**0.038**	**2.49**
Balkar	0.038	5.60
**Iberia_MN**	**0.038**	**3.98**
Spanish_North	0.037	4.91
**Motala_HG**	**0.037**	**4.13**
Belarusian	0.037	5.38
Spanish	0.037	5.72
Nogai	0.037	5.21

Ancient individuals/populations are shown in bold. MN: Middle Neolithic, LN: Late Neolithic, LBA: Late Bronze Age, MBA: Middle Bronze Age, EBA: Early Bronze Age, HG: Hunter-Gatherer, BA: Bronze Age. Populations/individuals with the largest values of *D* are shown.

S8. UPGMA tree

The UPGMA tree shows that GD13a clusters in the same branch as the Caucasus Hunter-Gatherers (Kotias and Satsurblia) (Fig. S8).

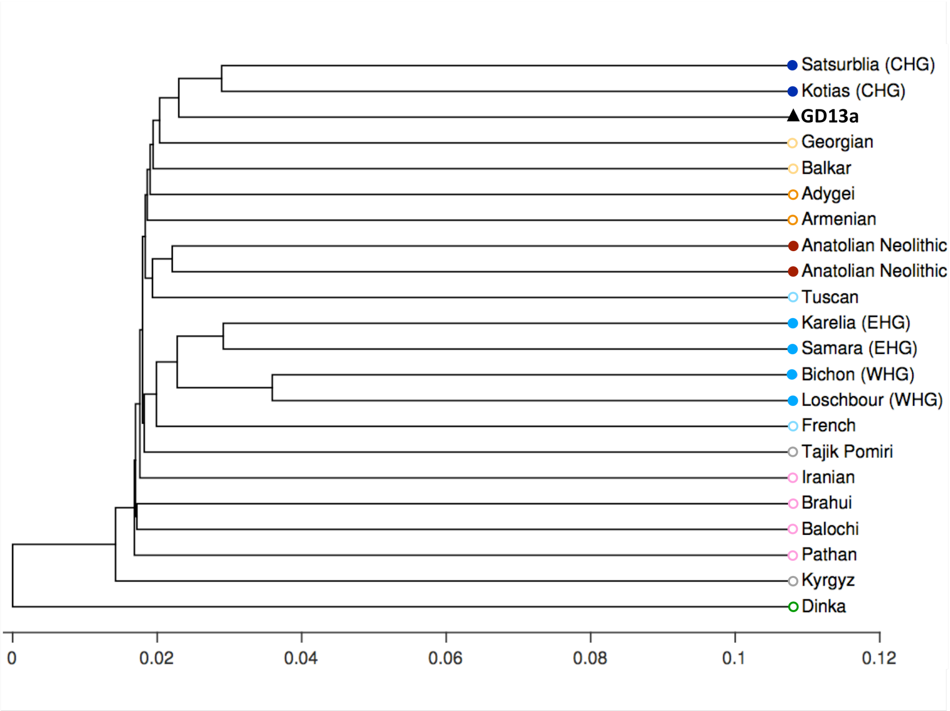

Fig. S8. UPGMA Tree, showing that GD13a clusters together with Caucasus Hunter Gatherers (CHG). EHG, Eastern Hunter Gatherers; WHG, Western Hunter Gatherers.

S9. Phenotypes of interest

Using the Hirisplex prediction model [12], GD13a was predicted to have brown eyes (p-value = 0.993) and dark (p-value=0.997), black (p-value=0.899) hair. This was confirmed using imputed genotypes. The eye-colour *HERC2* variant rs12913832 was assigned almost equal likelihoods of being homozygous for the ancestral allele (A; genotype probability = 0.501) and heterozygous (AG; genotype probability = 0.499). Given this result, and that the ancestral allele was observed (2-fold coverage) in the sample it is very likely that GD13a had at least one copy of the ancestral dominant allele associated with brown eyes. Using either state (homozygous ancestral or heterozygous) in the Hirisplex model and imposing a genotype probability cut-off of 0.9 for the other imputed genotypes, GD13a was predicted with the imputation approach to have dark (p-value $\geq$ 0.974), black (p-value $\geq$ 0.703) hair and brown eyes (p-value $\geq$ 0.952).

We did not observe the derived *SLC45A2* variant (rs16891982) associated with light skin pigmentation in GD13a (also supported by the imputed genotype) but did observe the derived *SLC24A5* variant (rs1426654) which is also associated with the same trait in modern populations. The imputed genotype for the latter suggests that this individual was heterozygous at this position (genotype probability > 0.999). Using either observed or imputed genotypes, GD13a did not show the most common variant of the *LCT* gene (rs4988235) associated with lactase persistence in Europeans (Table S5). We visually inspected the rs12913832 (HERC), rs1110400 (MC1R), and rs1426654 (SLC24A5) sites using the Integrative Genomics Viewer[13,14] as the observed alleles found at these sites may be the result of deaminated cytosine residues. We found that the alleles called at the rs12913832 and rs1110400 variant sites are greater than 10 bp from either end of the read. For the rs1426654 site at least one allele was 10 bp from either end of the read. This, combined with the

imputed genotypes, suggests that the alleles called at these position are unlikely to be the result of postmortem DNA damage which is more prevalent at read termini.

Table S5. Observed and imputed genotypes for GD13a at variant sites associated with phenotypes of interest.

Gene	Marker	Observed genotype	Coverage	Imputed genotype	Imputed Genotype probability
EXOC2	rs4959270	-	-	CA	> 0.999
HERC2	rs12913832	AA	2A	AA/GA	0.501/0.499
IRF4	rs12203592	-	-	CC	0.999
KITLG	rs12821256	-	-	TC	0.752
MC1R	N29insA	-	-	-	-
MC1R	rs1110400	TT	1T	TT	0.998
MC1R	rs11547464	-	-	GG	> 0.999
MC1R	rs1805005	-	-	GG	> 0.999
MC1R	rs1805006	-	-	CC	0.996
MC1R	rs1805007	CC	1C	CC	> 0.999
MC1R	rs1805008	CC	1C	CC	0.999
MC1R	rs2228479	-	-	GG	0.999
MC1R	rs885479	-	-	GG	0.913
MC1R	Y1520CH	-	-	-	-
MCIR	rs1805009	GG	1G	GG	0.999
OCA2	rs1800407	-	-	CC	0.985
PIGU/ASIP	rs2378249	-	-	AA	> 0.999
SLC24A4	rs12896399	GG	3G	GG	> 0.999
SLC24A4	rs2402130	GG	1G	GA	> 0.999
SLC45A2	rs16891982	CC	2C	CC	> 0.999
SLC45A2	rs28777	CC	1C	CC	0.999
TYR	rs1042602	CC	1C	CC	> 0.999
TYR	rs1393350	GG	3G	GG	> 0.999
TYRP1	rs683	CC	2C	CC	> 0.999
SLC24A5	rs1426654	AA	2A	AG	> 0.999
LCT	rs4988235	GG	2G	GG	> 0.999

S10. *D*-statistics show that Kotias and GD13a are possible surrogates for Ancestral North Indians

It has been proposed that modern Indians are a mixture of two ancestral components, an Ancestral North Indian (ANI) component related to modern West Eurasians and an Ancestral South Indian component related more distantly to the Onge, which has proved to be the best surrogate for it[15,16]. Meanwhile, Kotias has proven the best ancient surrogate for the ANI component[10]. We used *D*-statistics to formally assess the extent to which Kotias and GD13a relate to the ANI component in modern Indian populations. Overall, both genomes were plausible sources, with relatively little differences between them (Fig. S9, Table S6), even though Kotias was a marginally choice for a few comparisons. We also repeated the tests using Kharia, a sub-continental Indian population, to represent the ASI component (Fig. S10 and Table S7).

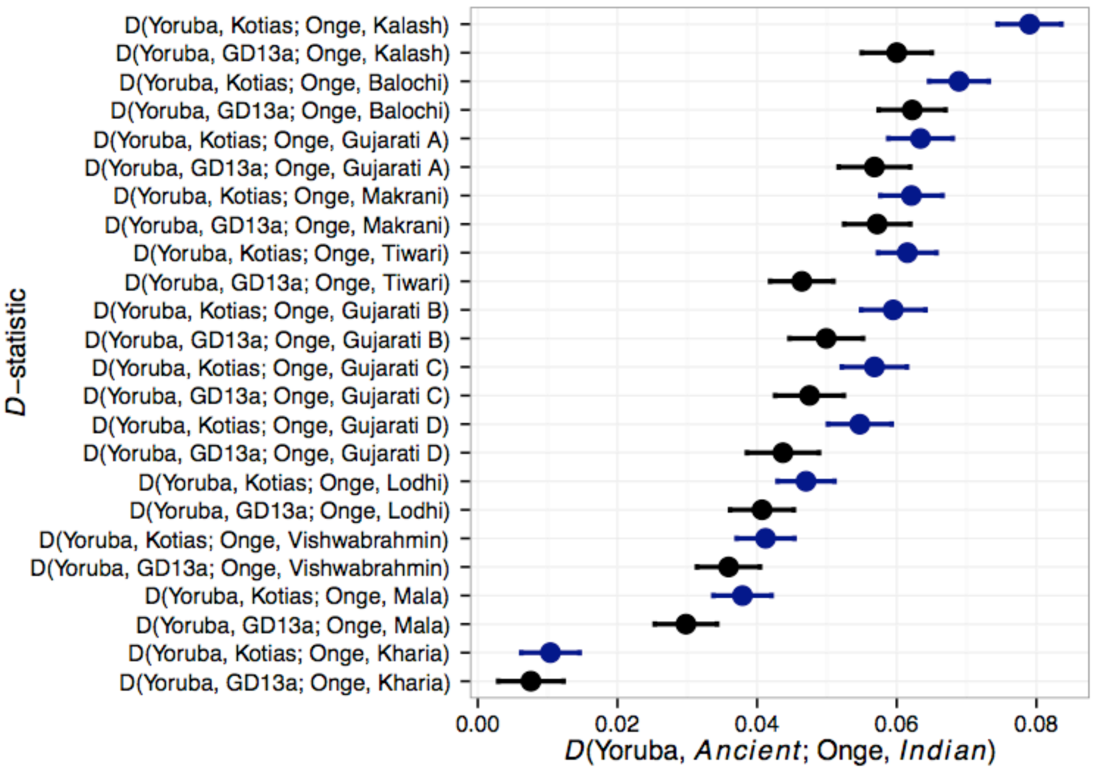

Fig. S9. *D*-statistics of the type *D*(Yoruba, *Ancient*; Onge, *South Asian*), where *Ancient* is represented by Kotias or GD13a, whereas *South Asian* is represented by Modern South Asian populations. Overall, both genomes were equally good proxies for ANI, even though Kotias was marginally better for a few comparisons (Kalash and Tiwari).

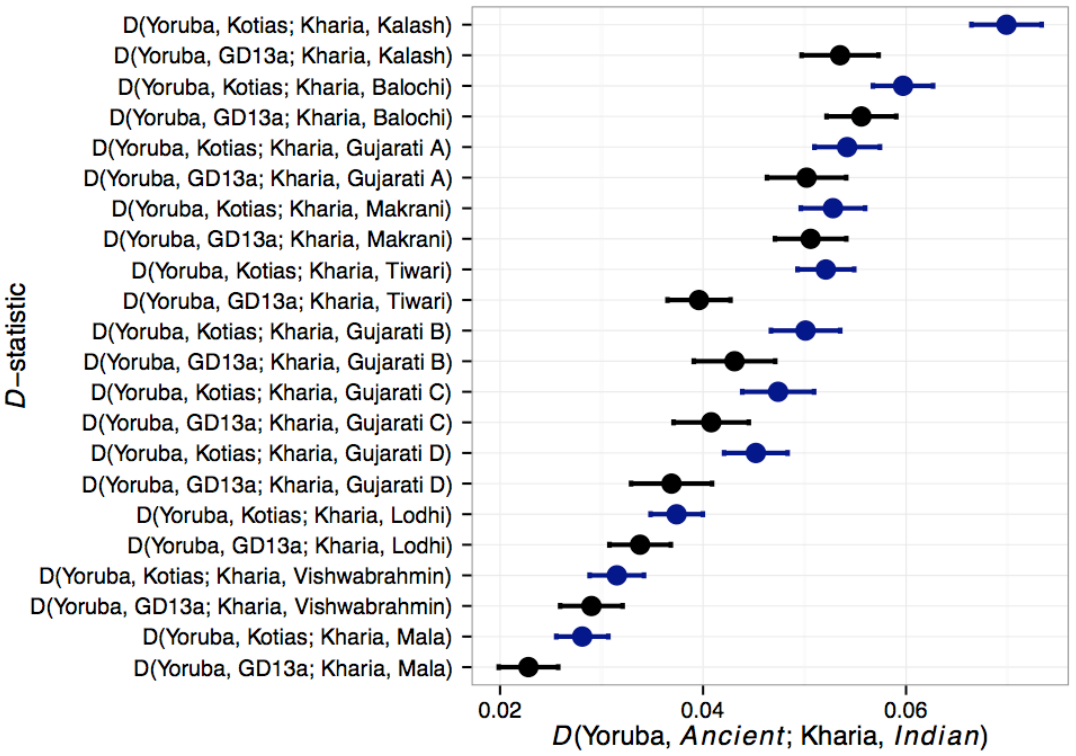

Fig. S10. *D*-statistics of the type *D*(Yoruba, *Ancient*; Kharia, *South Asian*), where *Ancient* is represented by Kotias or GD13a, whereas *South Asian* is represented by Modern South Asian populations. Overall, both genomes were equally good proxies for ANI, even though Kotias was marginally better for a few comparisons (Kalash and Tiwari).

Table S6: *D*-statistics of the form *D*(Yoruba, *Ancient*; Onge, *S. Asian*), where *Ancient* is either GD13a or Kotias, while *S. Asian* are different modern Indian and South Asian populations.

	D(Yoruba, GD13a; Onge, *S. Asian*)		*D*(Yoruba, Kotias; Onge, *S. Asian*)	
**Population**	***D*-statistic**	***Z***	***D*-statistic**	***Z***
Gujarati A	0.057	11.086	0.063	13.737
Gujarati B	0.050	9.462	0.060	12.943
Gujarati C	0.048	9.641	0.057	12.176
Gujarati D	0.044	8.455	0.055	11.992
Lodhi	0.041	8.914	0.047	11.531
Mala	0.030	6.673	0.038	9.092
Vishwabrahmin	0.036	7.946	0.041	9.952
Tiwari	0.046	10.236	0.062	14.701
Kharia	0.008	1.626	0.010	2.493
Kalash	0.060	11.935	0.079	17.256
Balochi	0.062	12.981	0.069	16.124
Makrani	0.057	12.060	0.062	13.959

Table S7: *D*-statistics of the form *D*(Yoruba, *Ancient*; Kalash, *S. Asian*), where *Ancient* is either GD13a or Kotias, while *S. Asian* are different modern Indian and South Asian populations.

	D(Yoruba, GD13a; Kharia, *S. Asian*)		*D*(Yoruba, Kotias; Kharia, *S. Asian*)	
**Population**	***D*-statistic**	***Z***	***D*-statistic**	***Z***
Gujarati A	0.050	12.856	0.054	16.79
Gujarati B	0.043	10.743	0.050	14.688
Gujarati C	0.041	11.016	0.047	13.398
Gujarati D	0.037	9.252	0.045	14.45
Lodhi	0.034	11.175	0.037	14.507
Mala	0.029	7.781	0.028	10.964

Vishwabrahmin	0.029	9.427	0.032	11.749
Tiwari	0.040	12.752	0.052	18.66
Kalash	0.054	14.101	0.070	20.194
Balochi	0.056	16.199	0.058	20.142
Makrani	0.051	14.399	0.053	16.662

S11. References:

1. Smith, P. E. L. in *Memorial Volume of the Vth International Congress of Iranian Art and Archaeology* **1,** 183–191
2. Young, T. C. & Smith, P. E. L. Research in the Prehistory of Central Western Iran. *Science* **153,** 386–391 (1966).
3. van Zeist, W., Smith, P. E. L., Palfenier-Vegter, R. M., Suwijn, M. & Casparie, W. A. An archaeobotanical study of Ganj Dareh Tepe, Iran. *Palaeohistoria* **26,** 201–224 (1984).
4. Kislev, M. E. in *People and culture in change: proceedings of the second symposium on Upper Palaeolithic, Mesolithic, and Neolithic Populations of Europe and the Mediterrenaien Basin (ed. Hershkovitz, I.)* 147–151 (B.A.R., 1989).
5. Riehl, S., Zeidi, M. & Conard, N. J. Emergence of Agriculture in the Foothills of the Zagros Mountains of Iran. *Science* **341,** 65–67 (2013).
6. Hesse, B. Slaughter Patterns and Domestication: The Beginnings of Pastoralism in Western Iran. *Man* **17,** 403–417 (1982).
7. Zeder, M. A. & Hesse, B. The Initial Domestication of Goats (Capra hircus) in the Zagros Mountains 10,000 Years Ago. *Science* **287,** 2254–2257 (2000).
8. Meiklejohn, C., Agelarakis, P. A., Smith, P. E. L. & Solecki, R. Artificial cranial deformation in the Proto-neolithic and Neolithic Near East and its possible origin : Evidence from four sites. *Paléorient* **18,** 83–98 (1992).
9. Haak, W. *et al.* Massive migration from the steppe was a source for Indo-European languages in Europe. *Nature* **522,** 207–211 (2015).
10. Jones, E. R. *et al.* Upper Palaeolithic genomes reveal deep roots of modern Eurasians. *Nat Commun* **6,** 8912 (2015).
11. R Development Core Team. *R: A Language and Environment for Statistical Computing*. (the R Foundation for Statistical Computing, 2001).

12. Walsh, S. *et al.* The HIrisPlex system for simultaneous prediction of hair and eye colour from DNA. *Forensic Sci. Int. Genet.* **7,** 98–115 (2013).

13. Robinson, J. T. *et al.* Integrative genomics viewer. *Nat Biotech* **29,** 24–26 (2011).

14. Thorvaldsdóttir, H., Robinson, J. T. & Mesirov, J. P. Integrative Genomics Viewer (IGV): high-performance genomics data visualization and exploration. *Brief Bioinform* **14,** 178–192 (2013).

15. Reich, D., Thangaraj, K., Patterson, N., Price, A. L. & Singh, L. Reconstructing Indian population history. *Nature* **461,** 489–494 (2009).

16. Moorjani, P. *et al.* Genetic Evidence for Recent Population Mixture in India. *The American Journal of Human Genetics* **93,** 422–438 (2013).

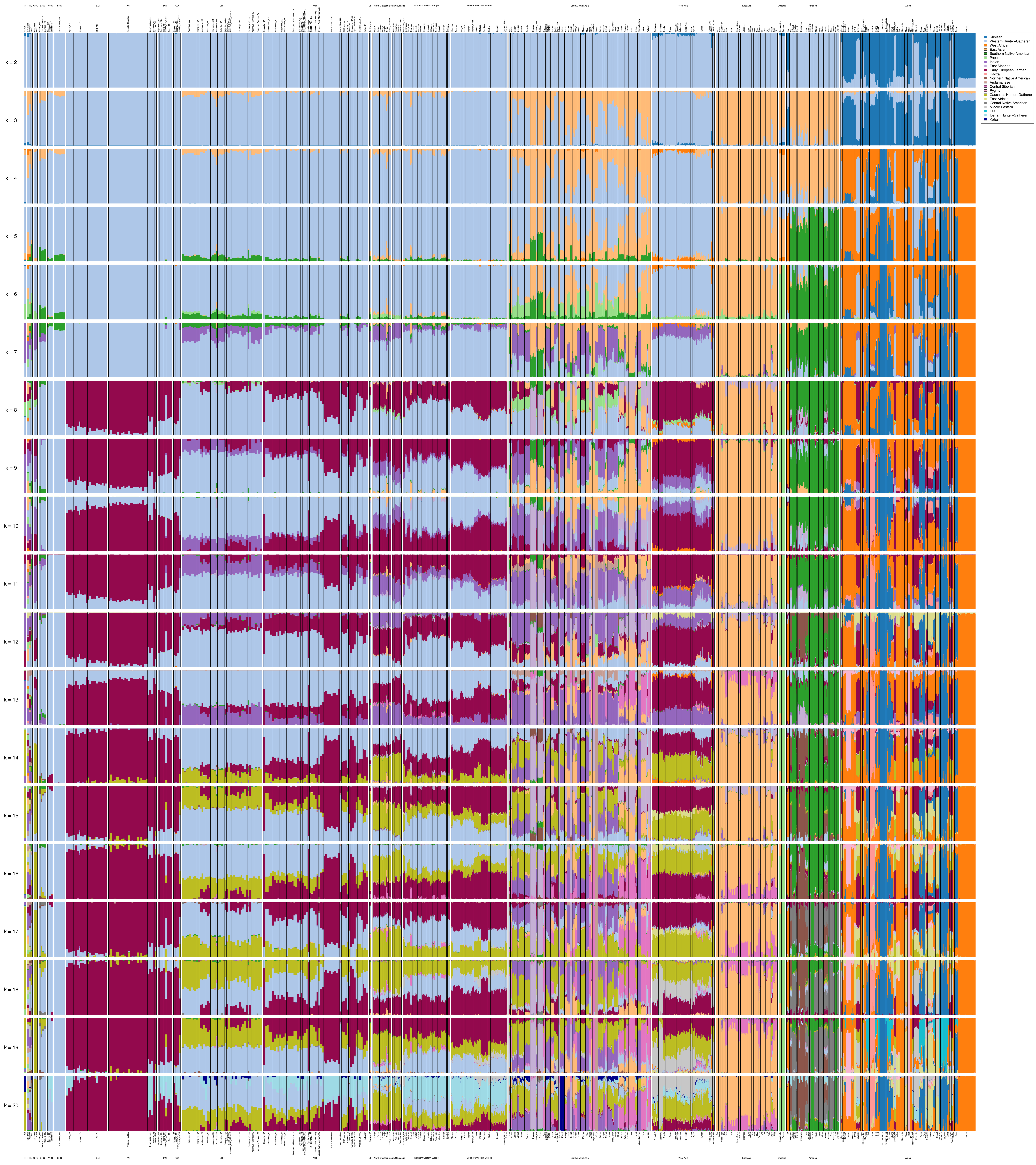

HG
PHG
CHG
EHG
WHG
SHG
EEF
AN
MN
CO
EBR
WBR
EIR
North Caucasus
South Caucasus
Northern/Eastern Europe
Southern/Western Europe
South/Central Asia
West Asia
East Asia
Oceania
America
Africa
k = 2
k = 3
k = 4
k = 5
k = 6
k = 7
k = 8
k = 9
k = 10
k = 11
k = 12
k = 13
k = 14
k = 15
k = 16
k = 17
k = 18
k = 19
k = 20
Khoisan
Western Hunter–Gatherer
West African
East Asian
Southern Native American
Papuan
Indian
East Siberian
Early European Farmer
Hadza
Northern Native American
Andamanese
Central Siberian
Pygmy
Caucasus Hunter–Gatherer
East African
Central Native American
Middle Eastern
Taa
Iberian Hunter–Gatherer
Kalash
